# Three-Dimensional Reconstruction Pre-Training as a Prior to Improve Robustness to Adversarial Attacks and Spurious Correlation

**DOI:** 10.3390/e26030258

**Published:** 2024-03-14

**Authors:** Yutaro Yamada, Fred Weiying Zhang, Yuval Kluger, Ilker Yildirim

**Affiliations:** 1 Department of Statistics & Data Science, Yale University, New Haven, CT 06511, USA; yutaro.yamada@yale.edu (Y.Y.); fred.zhang@yale.edu (F.W.Z.); 2 Department of Pathology, Yale University School of Medicine, New Haven, CT 06511, USA; yuval.kluger@yale.edu; 3 Department of Applied Mathematics, Yale University, New Haven, CT 06511, USA; 4 Foundations of Data Science Institute, Yale University, New Haven, CT 06511, USA; 5 Department of Psychology, Yale University, New Haven, CT 06511, USA; 6 Wu-Tsai Institute, Yale University, New Haven, CT 06511, USA

**Keywords:** robust vision, adversarial examples, 3D vision

## Abstract

Ensuring robustness of image classifiers against adversarial attacks and spurious correlation has been challenging. One of the most effective methods for adversarial robustness is a type of data augmentation that uses adversarial examples during training. Here, inspired by computational models of human vision, we explore a synthesis of this approach by leveraging a structured prior over image formation: the 3D geometry of objects and how it projects to images. We combine adversarial training with a weight initialization that implicitly encodes such a prior about 3D objects via 3D reconstruction pre-training. We evaluate our approach using two different datasets and compare it to alternative pre-training protocols that do not encode a prior about 3D shape. To systematically explore the effect of 3D pre-training, we introduce a novel dataset called Geon3D, which consists of simple shapes that nevertheless capture variation in multiple distinct dimensions of geometry. We find that while 3D reconstruction pre-training does not improve robustness for the simplest dataset setting, we consider (Geon3D on a clean background) that it improves upon adversarial training in more realistic (Geon3D with textured background and ShapeNet) conditions. We also find that 3D pre-training coupled with adversarial training improves the robustness to spurious correlations between shape and background textures. Furthermore, we show that the benefit of using 3D-based pre-training outperforms 2D-based pre-training on ShapeNet. We hope that these results encourage further investigation of the benefits of structured, 3D-based models of vision for adversarial robustness.

## 1. Introduction

Adversarial examples were first reported about a decade ago [1]. Despite tremendous research efforts since then, adversarial robustness remains perhaps the most important challenge to safe, real-world deployment of modern computer vision systems. Many proposals to defend against adversarial perturbations are later found to be broken [2]. A promising defense method that has withstood scrutiny is adversarial training [3]. Previous work extends adversarial training via surrogate-loss [4], using additional unlabelled data [5,6], or pre-training on more natural images [7]. However, recent work shows that adversarially trained image classifiers tend to rely on backgrounds, which makes models more sensitive to spurious correlations [8].

In this work, we turn to recent advances in 3D computer vision that incorporate prior knowledge of how 3D scenes are projected to 2D images via differentiable rendering (especially implicit neural representations [9,10]). The 3D reconstruction objective during pre-training implicitly encodes the prior over-3D scene structure (object shape and pose). We investigate how weight initialization via 3D reconstruction pre-training improves upon adversarial training in terms of robustness to both adversarial attacks and spurious correlation.

**Figure 1:**
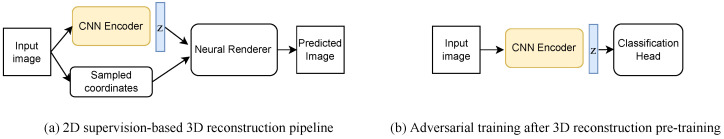
(**a**) A class of 3D reconstruction models we are interested in is presented, where a CNN encoder is used to condition the 3D reconstruction model on shape features of 2D input images. (**b**) To leverage 3D-based pre-training, we extract the weights from the CNN encoder that is pre-trained on 3D reconstruction and use them as initialization for adversarial training on 2D rendered images of 3D objects. The goal of this paper is to investigate the effect of 3D reconstruction pre-training of these image encoders on adversarial robustness.

To do so, we consider recent 3D reconstruction models that are equipped with an image encoder based on Convolutional Neural Networks (CNNs). The goal of such an image encoder is to produce efficient representations for 3D reconstruction, and therefore, it is expected to encode an implicit prior of 3D scenes, as summarized in Figure 1.

Our proposal is inspired by probabilistic models of human vision, that emphasize (in addition to uncertainty) the richness of perception in terms of 3D geometry, including object shape and pose. This ability to make inferences about the underlying scene structure from input images—also known as analysis-by-synthesis—is thought to be critical for the robustness of biological vision [11,12]. Our work leaves the role of proper uncertainty quantification (via Bayesian inference) for improving robustness to adversarial and spurious correlation attacks as future work and instead focuses on implicitly encoding prior knowledge about inferring 3D geometry.

Standard benchmark datasets for adversarial robustness of image classifiers—e.g., MNIST, CIFAR-10 and Tiny-ImageNet—are not suitable to address our question. These datasets are not designed to be useful for 3D reconstruction tasks. To understand the interplay between encoding prior knowledge about 3D geometry via pre-training and the performance of adversarial training, we introduce *Geon3D*—a novel dataset comprising simple yet realistic shape variations, derived from the human object recognition hypothesis called *geon theory* [13].

Using Geon3D as a bridge from simple objects to more complex real shape objects like ShapeNet, we systematically perform experiments varying the complexity of the shape dataset. We first find that 3D-based pre-training does not improve the performance of adversarial training in the simplest shape dataset we consider (Geon3D with black background). However, when in a more realistic variation of Geon3D with textured backgrounds, we find 3D-based pre-training strengthens $L_{\infty }$-based adversarial training. When we introduce spurious correlation between shape and background, 3D-based pre-training outperforms vanilla adversarial training for both $L_{\infty }$ and $L_{2}$ threat models in terms of robustness to spurious correlation. We further confirm that this trend continues to hold for more complicated shape objects, namely ShapeNet dataset [14]. Crucially, we show that the benefit of 3D-based pre-training outperforms 2D-based pre-training on ShapeNet. While our study is limited to shape datasets, as 3D reconstruction techniques improve to deal with increasingly more realistic and complicated settings, we hope our study serves as a first step towards better understanding the relationship between 3D vision and adversarial robustness.

## 2. Related Work

**Pre-training for adversarial training**. Ref. [7] proposes pre-training to improve adversarial robustness, but their work focuses on classification-based pre-training by introducing more natural images. In contrast, our work uses pre-training to encode a prior about 3D object shape and pose. In addition to pre-training, some work explores using additional data. Carmon et al. [5], Alayrac et al. [6] propose using unlabelled data, where they improve adversarial robustness by training models on CIFAR-10 and unlabelled data from the 80 Million Tiny Images dataset. These works are orthogonal to ours, since our work specifically looks to incorporate priors about 3D geometry.

**Shape bias to induce robustness**. A recent line of work explores methods to increase *shape bias* as a way to make neural network models more robust to image perturbations [15,16,17]. A notable example is given by [15], who proposes to train a model on Stylized-ImageNet (SIN), which is created by imposing various painting styles onto images from ImageNet [18]. Unlike these studies, which indirectly tackle shape bias by reducing the reliance on texture, our work induces shape bias directly into image classifiers, via 3D reconstruction pre-training. Recent studies show that generative classifiers based on text-to-image diffusion models [19] achieve human-level shape bias [20]. Our research is in line with this field of study, but instead of using text-to-image generative models, we focus on employing 3D generative models.

**Three-dimensional datasets**. Geon3D is smaller in scale and less complex in shape variation relative to some of the existing 3D model datasets, including ShapeNet [14], ModelNet [21], OASIS [22] and Rel3D [23]. These datasets have been instrumental for recent advances in 3D computer vision models (e.g., [24,25]). As we demonstrate in this work, Geon3D allows us to systematically study the relationship between 3D-based pre-training and adversarial training by varying the complexity of the dataset, bridging toy datasets to more realistic datasets such as ShapeNet.

**Other types of robustness**. There have been many studies that attempt to improve robustness of vision models and, more generally, to align model prediction with human judgement. Existing work has attempted to leverage features such as low-frequency features [26,27] and biologically constrained Gabor filters [28]. Ref. [29] introduces a common corruption benchmark for ImageNet models. Ref. [30] shows that latest-vision transformer models start to close the gap between human and machine vision in terms of robustness, while room for improvement still exists.

## 3. Three-Dimensional Reconstruction as Pre-Training

Recently, there has been significant progress in learning-based approaches to 3D reconstruction, where the data representation can be classified into voxels [31,32], point clouds [33,34], mesh [35,36] and neural implicit representations [9,10,25,37]. In this paper, we are interested in methods that can be used to pre-train an image encoder so that we can use the weights of the pre-trained image encoder as initialization for adversarial training of image classifiers. For this purpose, we avoid 3D reconstruction models based on voxels, point clouds and 3D meshes, since they are not easily transferable to image classification settings. Luckily, neural implicit representation allows the community to develop a class of models that only requires 2D supervision. Neural implicit representation is built upon the idea that shape can be represented by the level sets of a function $f:R^{3}\to R$, and *f* is approximated by neural networks.

Specifically, we use two recent 3D reconstruction models, Differentiable Volumetric Rendering (**DVR**) [24] and **pixelNeRF** [38], both of which consist of a CNN-based image encoder and a differentiable neural rendering module. While implicit representation of 3D objects is completed with a neural network-based rendering module in the 3D reconstruction model, we hypothesize that an image encoder of the 3D reconstruction model is biased towards producing an encoded representation that is useful for 3D geometry understanding. The main object of our study is to see to what extent we can leverage 3D reconstruction pre-training to improve adversarial robustness. We take the encoder of the trained 3D reconstruction model and attach a classification head and then finetune, which is described in Figure 1.

### Problem Setup for 3D Reconstruction

Both DVR and pixelNeRF are based on neural implicit representations. DVR learns the occupancy field via neural networks and represents objects via the zero-level set, which is found via ray-marching. The points corresponding to the zero-level are used to query a texture network, which produces RGB values as rendered images. The image encoder of DVR is used to condition the occupancy network and texture network. PixelNeRF is based on NeRF, which learns radiance field via a neural network. Given a spatial point and viewing direction, the radiance field returns the density and RGB color. PixelNeRF additionally conditions NeRF by the local image features produced by the image encoder. The radiance field can then be rendered by volumetric rendering. We note that only DVR requires object masks, and pixelNeRF can be trained fully based on 2D images and camera matrices. For more details on the problem setup and training, we refer the readers to the Appendix C.

## 4. Geon3D Benchmark

The concept of *geons*—or *geometric ions*—was originally introduced by Biederman as the building block for their Recognition-by-Components (RBC) Theory [13]. The RBC theory argues that human shape perception segments an object at regions of sharp concavity, modeling an object as a composition of geons—a subset of generalized cylinders [39]. Similar to generalized cylinders, each geon is defined by its axis function, cross-section shape and sweep function. To reduce the possible set of generalized cylinders, Biederman considered the properties of the human visual system. He noted that the human visual system is better at distinguishing between straight and curved lines than at estimating curvature; detecting parallelism than estimating the angle between lines; and distinguishing between vertex types such as an arrow, Y and L-junction [40].

This paper is not focused on the validity of the RBC theory. Instead, we wish to build upon the way in which Biederman characterized these geons. Biederman proposed using two to four values to characterize each feature of the geons. Namely, the axis can be straight or curved; the shape of cross section can be straight-edged or curved-edged; the sweep function can be constant, monotonically increasing/decreasing, monotonically increasing and then decreasing (i.e., expand and contract), or monotonically decreasing and then increasing (i.e., contract and expand); the termination can be truncated, end in a point, or end as a curved surface. A summary of these dimensions is given in Table 1.

**Figure 2:**
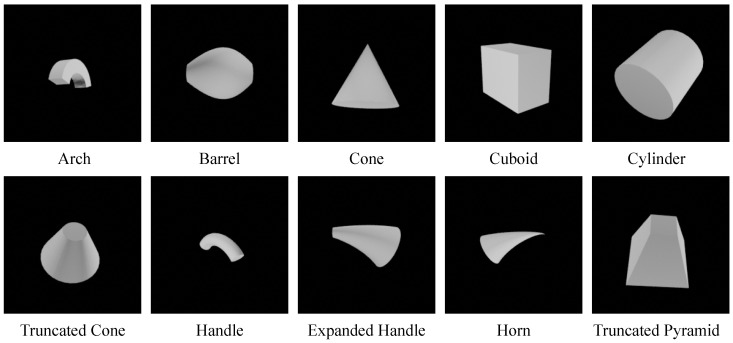
Examples of 10 geon categories from Geon3D. The full list of 40 geons we construct (Geon3D-40) is provided in the Appendix A.

Representative geon classes are shown in Figure 2. For example, the “Arch” class is uniquely characterized by its curved axis, straight-edged cross section, constant sweep function and truncated termination. These values of geon features are *nonaccidental*—we can determine whether the axis is straight or curved from almost any viewpoint, except for a few *accidental* cases. For instance, an arch-like curve in the 3D space is perceived as a straight line only when the viewpoint is aligned in a way that the curvature vanishes. We list similar geon categories, where only a single feature differs in Table 2.

### 4.1. Data Preparation

We construct each geon using Blender (https://www.blender.org, accessed on 1 January 2022)—an open-source 3D computer graphics software [41].

An advantage of geons over other geometric primitives such as superquadrics [42] is that the shape categorization of geons is qualitative rather than quantitative. That is, each geon feature, such as the main axis being curved or not, is explicitly categorical, whereas each deformation of shape is continuous and does not change geon features that define each geon category. Thus, each geon category affords a high degree of in-class shape deformation, as long as the four defining features of each shape class remains the same. Such flexibility allows us to construct a number of different 3D model instances for each geon class by expanding or shrinking the object along the *x*, *y*, or *z*-axis. In our experiments, for each axis, we evenly sample the 11 scaling parameters from the interval [0.5, …, 1.5] with a step size 0.1, resulting in 1331 model instances for each geon category.

### 4.2. Rendering and Data Splits

We randomly sample 50 camera positions from a sphere with the object at the origin. For each model instance, 50 images are rendered using these camera positions with a resolution of 224 × 224. We then split the data into train/validation/test with a ratio of 8:1:1 using model instance ids, where each instance id corresponds to the scaling parameters described above. For more details of data preparation, see the Appendix A.

## 5. General Methods for Experiments

### 5.1. Pre-Training

We use DVR and pixelNerf as our 3D reconstruction models. During 3D reconstruction pre-training, we first sample object instance ids of batch size, and then randomly sample a single view for each object instance to form a mini-batch, following the community convention of 3D reconstruction training. For the image encoder of 3D reconstruction models, we use ResNet18, which is expected to encode shape and category information during training. In the following Geon3D and ShapeNet experiments, we focus on the pre-training method that performs better 3D reconstruction on the respective dataset (e.g., DVR for Geon3D and pixelNeRF for ShapeNet).

### 5.2. Adversarial Training

We used the Python package (https://github.com/MadryLab/robustness (accessed on 1 January 2022)) to perform adversarial training (AT) [3]. Throughout the experiments in this paper, we study a threat model where the adversary is constrained to $L_{p}$-bounded perturbations, where we use p=∞ and p=2. We consider the white-box setting, where we assume that the adversary has complete knowledge of the model and its parameters. For AT-$L_{2}$ training, we train our models via Projected Gradient Decent (PGD) [3] for 60 epochs with the batch size of 50, the attack steps of 7, the perturbation budget ϵ of 1.0, and the attack learning rate of 0.2. For AT-$L_{\infty }$ training, we train our models for 60 epochs with the batch size of 100, the attack steps of 7, the perturbation budget of 0.05, and the attack learning rate of 0.01. We use the best PGD step as an adversarial example during training. We use ResNet-18 [43] as our architecture throughout our experiments.

### 5.3. Evaluation

It is notoriously difficult to correctly evaluate adversarial robustness [2]. The attack based on Projected Gradient Descent (PGD) ([3]) is widely used, but many defense methods are later found to be broken partly because PGD requires careful parameter tuning to be a reliable attack. To mitigate these issues, ref. [44] proposes AutoAttack, which is an ensemble of four strong, diverse attacks: two extensions of PGD, the white-box fast adaptive boundary (FAB) attack [45], and the black-box Square Attack [46]. We use AutoAttack with the default parameter setting for both $L_{\infty }$ and $L_{2}$ robustness evaluation throughout our experiments.

### 5.4. Additional Training Details

We used Tesla V100 GPUs for all of our experiments. DVR 3D reconstruction training takes roughly about 1.5 days on a single GPU. The hyperparameters for adversarial training, described in the main paper, were chosen by monitoring the model convergence on the validation set. All the other results are from a single training run and a single evaluation run.

#### 5.4.1. DVR

We used the code (https://github.com/autonomousvision/differentiable_volumetric_rendering, accessed on 1 January 2022) open-sourced by [24]. We followed the default hyperparameters recommended by [24] for 3D reconstruction training, with the exception of batch size, which we set as 32 to fit into a single GPU memory.

#### 5.4.2. PixelNeRF

We use the code (https://github.com/sxyu/pixel-nerf, accessed on 1 January 2022) open-sourced by the original authors [38].

#### 5.4.3. AE and VAE

We use the code (https://pytorch-lightning-bolts.readthedocs.io/en/latest/models/autoencoders.html, accessed on 1 January 2022) from pytorch-lightning bolts to train AE and VAE on ShapeNet. Both the encoder and decoder are based on ResNet18.

#### 5.4.4. Dataset

For training Geon3D image classifiers, we center and re-scale the color values of Geon3D with μ=[0.485,0.456,0.406] and σ=[0.229,0.224,0.225], which is estimated from ImageNet. We construct the 40 3D model instances as well as the whole training data in Blender. We then normalize the object bounding box to a unit cube, which is represented as 1.0_1.0_1.0 in the dataset folder.

#### 5.4.5. Background Textures

We used the following label-to-texture class mapping: {0: ‘zigzagged’; 1: ‘banded’; 2: ‘wrinkled’; 3: ‘striped’; 4: ‘grid’; 5: ‘polka-dotted’; 6: ‘chequered’; 7: ‘blotchy’; 8: ‘lacelike’; 9: ‘crystalline’}. For the distributional shift experiment, we used the following mapping: {0: ‘crystalline’; 1: ‘zigzagged’; 2: ‘banded’; 3: ‘wrinkled’; 4: ‘striped’; 5: ‘grid’; 6: ‘polka-dotted’; 7: ‘chequered’; 8: ‘blotchy’; 9: ‘lacelike’}. The DTD data are licensed under the Creative Commons Attribution 4.0 License (https://creativecommons.org/licenses/by/4.0/, https://www.tensorflow.org/datasets/catalog/dtd, accessed on 1 January 2022).The texture images are randomly sampled from the DTD data. We used a variety of textures with the same style for each category. Specifically, DTD has 47 texture categories in total, and there are 120 texture images for each category. We pick 10 categories for Geon3D, so the stimuli included as backgrounds are sampled from 1200 texture images for Geon3D.

## 6. Experiments Using Geon3D

In this section, we will use the Geon3D shapes to create three increasingly more challenging datasets: (*i*) Geon3D with clean background (“Black Background”), (*ii*) Geon3D with randomly assigned textured backgrounds (“Textured Background”) and (*iii*) Geon3D with correlated textured backgrounds, which introduces spurious correlations between background textures and categories (“Spurious Correlations”). For simplicity, we focus on 10 representative geon categories (instead of the full 40 categories) and call it the Geon3D dataset. The dataset for adversarial training is a subset of the Geon3D data we used for 3D reconstruction pre-training. Specifically, we sample 10,000/1000/1000 images for training, validation, and test sets, respectively. We ensure that we sub-sample each split from the original train/val/test splits of Geon3D so that there is no data leakage from pre-training to adversarial training.

### 6.1. Adversarial Robustness

#### 6.1.1. Setup

**Figure 3:**
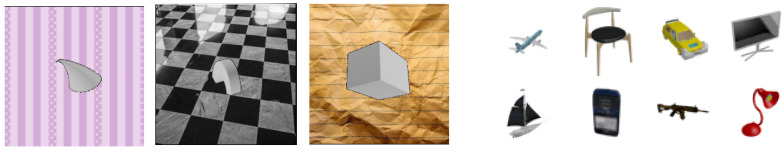
(**Left**) Example images from Geon3D with textured backgrounds. (**Right**) Example images from ShapeNet.

We start from the simplest setting: Geon3D with black background. We then vary the complexity of the experimental setting by introducing background textures to the dataset. Specifically, we replace each black background of Geon3D with a random texture image out of 10 texture categories chosen from the Describable Textures Dataset (DTD) [47]. Example images from this Geon3D Textured Background dataset can be seen in Figure 3 (Left). These two datasets allow us to analyze the effect of 3D reconstruction pre-training as a function of dataset (in particular, background) complexity.

#### 6.1.2. Results

**Figure 4:**
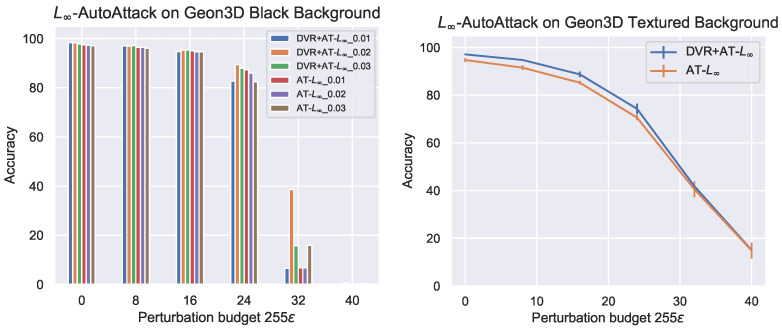
Adversarial robustness of vanilla adversarial training (AT) and 3D-based pre-training with increasing perturbation budget for $L_{\infty }$ threat model on Geon3D with black and textured backgrounds. DVR stands for Differentiable Volume Rendering. For textured backgrounds, we perform our experiments three times with different random initialization for the classification linear layer, where we use DVR-pretrained ResNet-18 and ImageNet-pretrained ResNet-18 for the main backbone. We report the mean and standard deviation over these three runs. For Black Background, we run AT with different attack learning rates (0.1, 0.2 and 0.3) and report its adversarial accuracy. Here, we use the adversarial perturbation budget of 0.05, which corresponds to 12.75 on the *x*-axis, for both textured backgrounds and black backgrounds during adversarial training. Between the simplest setting of Geon3D with black background and Geon3D with textured background, we observe that the effect of 3D reconstruction pre-training (DVR) emerges only under the latter. The perturbation budget during adversarial training is 0.05, which corresponds to 12.75 on the *x*-axis.

**Figure 5:**
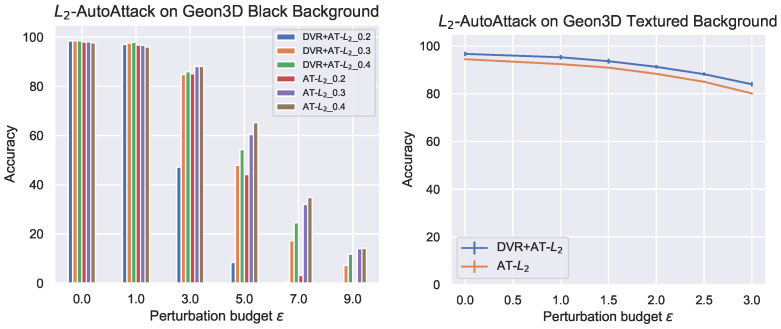
Adversarial robustness of AT and DVR+AT with increasing perturbation budget for $L_{2}$ threat models on Geon3D. For $L_{2}$ textured backgrounds, we perform our experiments three times with different random initialization for the classification linear layer, where we use DVR-pretrained ResNet-18 and ImageNet-pretrained ResNet-18 for the main backbone. We report the mean and standard deviation over these three runs, where we see a small variance for $AT-L_{2}$. For $L_{2}$ Black Background, we run AT with different attack learning rates (0.2, 0.3 and 0.4) and report its adversarial accuracy. Here, we use the adversarial perturbation budget of 3.0 for textured backgrounds and 1.0 for black backgrounds during adversarial training. In the aggregate, 3D pre-training does not improve, and in fact lowers, the performance of AT for black backgrounds. However, similar to the $L_{\infty }$ case, we continue to see the trend that 3D-based pre-training helps more for textured backgrounds.

Seen in Figure 4 are the results of adversarial robustness evaluation for $L_{\infty }$ threat models. For the black background, DVR+AT slightly outperforms AT for ϵ=8/255, but as the the perturbation budget becomes large, AT outperforms DVR+AT. However, for the textured background, DVR+AT consistently outperforms vanilla AT across all perturbation budgets. Figure 5 shows the results of adversarial robustness with $L_{2}$ threat models. On both the black and textured background settings, we find that AT is on average, across all perturbation budgets, more robust than DVR+AT. However, consistent with the $L_{\infty }$ results, we see that DVR+AT better performs on the more complex textured background setting, slightly outperforming AT for small perturbation budgets.

### 6.2. Robustness to Spurious Correlations between Shape and Background

#### 6.2.1. Setup

Recent work [8] shows that adversarially trained image classifiers tend to rely on backgrounds rather than objects. Can 3D pre-training help mitigate such reliance of backgrounds for adversarial training? Here, we test whether 3D-based pre-training, which directly targets shape features (e.g., scene geometry that causes pixel intensity values only on the foreground object), improves over vanilla AT in terms of robustness towards spurious correlation that is created by backgrounds.

To do this, we create a new variant of Geon3D, where we choose 10 texture categories from DTD and introduce spurious correlations between shape category and textured background class (i.e., each shape category is paired with one texture class). During 3D pre-training, we feed this dataset (referred to as “Correlated Texture”) to the image encoder of the 3D reconstruction model. Adversarial training of all models is also performed using this dataset. Therefore, during adversarial training, a model can pick up classification signals from both the shape of the geon as well as the background texture. To evaluate whether or not 3D pre-training helps models ignore spurious correlations more effectively, we prepare a test set that breaks the correlation between shape category and background texture class by cyclically shifting the texture class from *i* to i+1 for i=0,…,9, where the class 10 is mapped to the class 0. This design is inspired by [15]; however, in our case, a distributional shift from training to test set is designed to isolate out and directly measure the effect of 3D prior by fully disentangling the contributions of texture and shape.

#### 6.2.2. Results

We note that in this section, we do not perform adversarial attacks, but simply evaluate classification accuracy of all models on the newly constructed test set that breaks the correlation between textures and shape, as described above.

The results are shown in Table 3. We find that regardless of the perturbation set, DVR+AT outperforms AT, by a large margin in the case of $L_{2}$ and still substantially for $L_{\infty }$. Together, these results suggest that we can view 3D-based pre-training as a way to bias models to prefer shape features, even in the presence of strong, spurious correlations.

#### 6.2.3. Summary

We have varied the background texture and texture-shape correlation of Geon3D and measured how such variation affects the relationship between 3D-based pre-training and adversarial robustness. Our results with Geon3D so far suggest that the benefit of 3D-based pre-training emerges in the setting of spurious correlation.

## 7. Experiments Using More Complex Objects: ShapeNet

### 7.1. Setup

We use ShapeNet [14] to evaluate the effect of 3D reconstruction pre-training on adversarial robustness under a shape distribution that is significantly more complex than Geon3D. Example images from ShapeNet are shown in Figure 3. We use the 13 most densely sampled shape categories from ShapeNet, as is commonly used in 3D reconstruction benchmarks. We perform 3D-based pre-training using the pixelNerf (PxN) model, which performs the basic task of 3D reconstruction more accurately than the DVR model on the ShapeNet dataset [38]. However, we note that we find similar results using DVR as the pre-training architecture (see Figure 7). After 3D-based pre-training, we sub-sample 130,000/13,000/13,000 images as training/validation/test splits for adversarial training. We also ensure that object instances that are used for 3D reconstruction do not overlap with validation and test splits for adversarial training, so that there is no data leakage from pre-training.

### 7.2. Results

**Figure 6:**
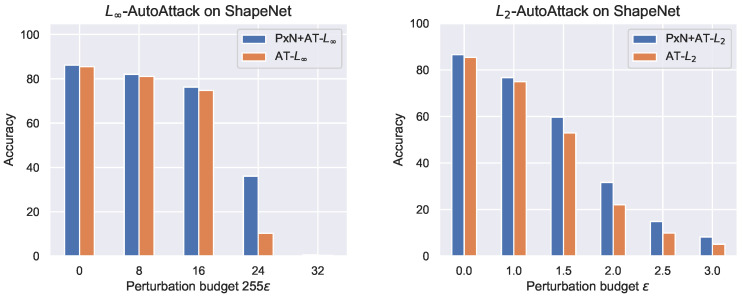
Adversarial robustness of AT and PxN+AT with increasing perturbation budget for ShapeNet. PxN stands for pixelNeRF. We see that 3D reconstruction pre-training (PxN+AT) improves over vanilla adversarial training (AT) for both $L_{\infty }$ and $L_{2}$ across all perturbation budgets. The perturbation budget during adversarial training is 0.05, which corresponds to 12.75 on the *x*-axis for $L_{\infty }$ and 1.0 for $L_{2}$ threat models.

Figure 6 shows the results of adversarial robustness on ShapeNet. In contrast to previous results, we can see that for both $L_{\infty }$ and $L_{2}$ threat models, 3D-based pre-training (PxN+AT) improves over vanilla AT, across the entire range of perturbation budgets. This suggests that as we increase the complexity of object shapes, the 3D-based pre-training more consistently yields better robustness.

### 7.3. Adversarial Robustness on ShapeNet

**Figure 7:**
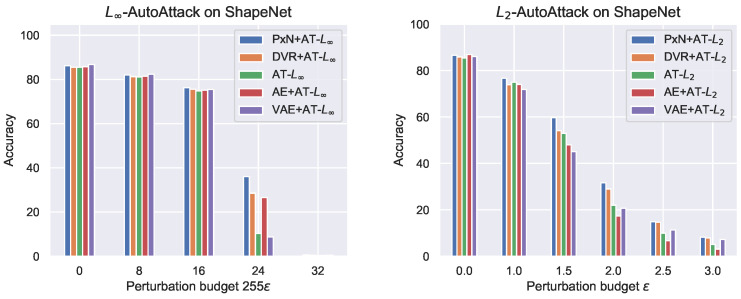
Adversarial robustness comparison between PxN+AT, DVR+AT, AE+AT, VAE+AT and AT for both $L_{\infty }$ and $L_{2}$ threat models with increasing perturbation budget ϵ on ShapeNet. The perturbation budget during adversarial training is 0.05, which corresponds to 12.75 on the *x*-axis for $L_{\infty }$ and 1.0 for $L_{2}$ threat models.

In Figure 7, we show additional results of adversarial robustness for both $L_{\infty }$ and $L_{2}$ threat models. In addition to PxN+AT, we include DVR+AT. We also include AE+AT and VAE+AT across the perturbations we tested. We see that 3D-based pre-training (PxN+AT and DVR+AT) outperforms 2D-based pre-training (AE+AT and VAE+AT) as we increase the magnitude of the perturbations ϵ.

**Figure 8:**
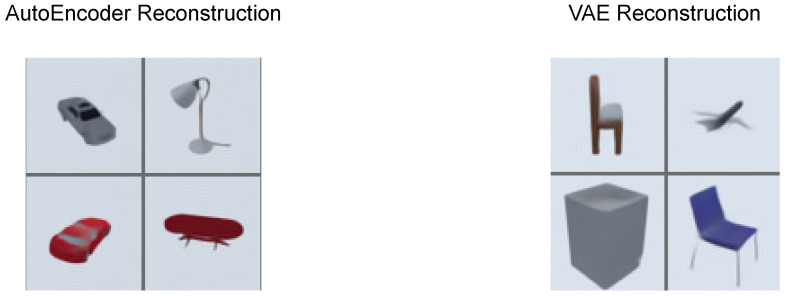
Reconstructed ShapeNet images. (**Left**) AutoEncoder, (**Right**) VAE.

Seen in Figure 8 are the reconstructed images of AutoEncoder and Variational AutoEncoder (VAE).

## 8. Limitations and Discussion

The advantage of Geon3D over other datasets lies in its simplicity, which makes it easier to isolate the effect of 3D shape features. This simplicity is beneficial for future research aimed at examining the relationship between model behavior and 3D shape features. However, there are limitations as well. In this paper, we view 3D reconstruction as a pre-training task that provides better weight initialization in the form of a 3D object prior. The robustness gained from such a 3D prior is necessarily constrained by the capability of the underlying 3D reconstruction models. We studied only one form of causal, thus by definition, robust set of features (3D shape and pose); future work should consider incorporating priors based on other causal variables, such as the physical properties of objects. We studied only one way to induce such a prior (via pre-training); future work should explore other ways in which explicit robust properties can be integrated to AT. Moreover, we focus on the aspect of structured representation of human cognition. Future work should also explore how uncertainty representation of human cognition can play a part in adversarial robustness. Finally, future work should understand why 3D pre-training is not helpful for the simplest data setting studied here.

## 9. Conclusions

We investigated how 3D-based pre-training can affect robust accuracy of adversarial training. We start from the simplest setting: Geon3D with black background. In this case, 3D-based pre-training does not improve vanilla adversarial training. However, we find that 3D-based pre-training improves over adversarial training under more complex data distributions, including the ShapeNet objects. Importantly, 3D-based pre-training outperforms 2D-based pre-training methods that otherwise receive identical training procedures. We hope that these results motivate further exploration of structured 3D-based models of vision for addressing adversarial robustness.

## Figures and Tables

**Table 1:** Latent features of Geons. S: straight; C: curved; Co: constant; M: monotonic; EC: expand and contract; CE: contract and expand; T: truncated; P: end in a point; CS: end as a curved surface.

Feature	Values
Axis	S, C
Cross-section	S, C
Sweep function	Co, M, EC, CE
Termination	T, P, CS

**Table 2:** Similar geon categories, where only a single feature differs out of four shape features. “T.” stands for “Truncated”. “E.” stands for “Expanded”.

Geon Category	Difference
Cone vs. Horn	Axis
Handle vs. Arch	Cross-Section
Cuboid vs. Cyllinder	Cross-Section
T. Pyramid vs. T. Cone	Cross-Section
Cuboid vs. Pyramid	Sweep function
Barrel vs. T. Cone	Sweep function
Horn vs. E. Handle	Termination

**Table 3:** Accuracy of adversarially trained models against distributional shift in backgrounds. Here, all models are trained on Geon3D Correlated Textured (with background textures correlated with shape categories) and evaluated on a test set where we break this correlation. We see that for both $L_{\infty }$ and $L_{2}$, pre-training using DVR biases the models to prefer shape features to textures. Moreover, the difference between two threat models of vanilla AT suggests that AT-$L_{2}$ prefers texture features, while AT-$L_{\infty }$ prefers shape features.

AT-$L_{2}$	DVR+AT-$L_{2}$	AT-$L_{\infty }$	DVR+AT-$L_{\infty }$
10.8	**35.6**	79.0	**84.20**

**Figure A1:**
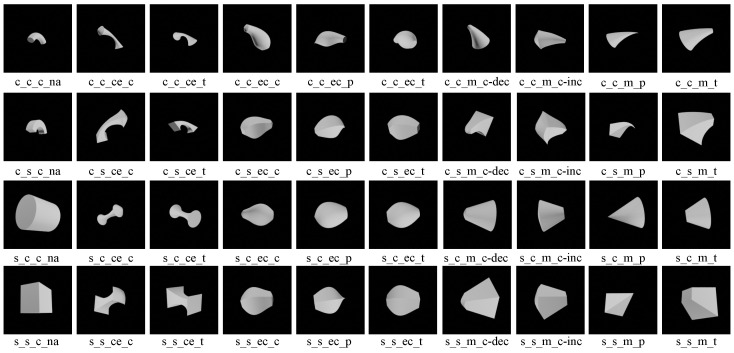
The list of 40 geons we constructed.

**Figure A2:**
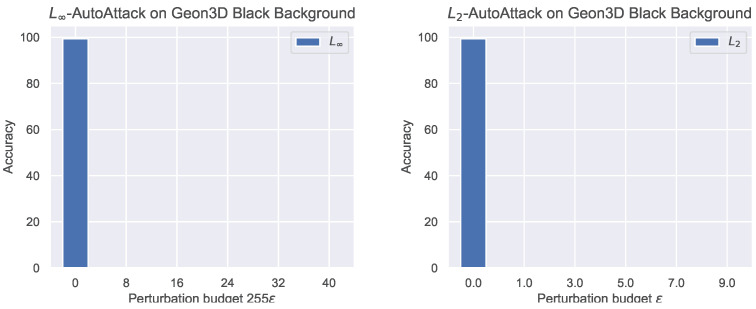
Adversarial robustness of 3D pre-trained ResNet-18 for both $L_{\infty }$ and $L_{2}$ threat models with increasing perturbation budget ϵ on Geon3D with black backgrounds.

## Data Availability

The datasets generated during and/or analyzed during the current study are available in the google drive repository through https://drive.google.com/file/d/1v5XwO-QrnB_j9XhJJl4c7d7hMQf-v6gq/view?usp=drive_link (accessed on 1 January 2022).

## Appendix A. Dataset

A line of work in psychophysics of human visual cognition has argued that the visual system exploits certain types of shape features in inferring 3D structure and geometry. In Geon3D, by treating these shape features as the dimensions of variation, we model 40 classes of 3D objects, and render them from random viewpoints, resulting in an image set and their corresponding camera matrices. Upon publication, we will make the Geon3D dataset publicly available. Examples of Geon3D are shown in Figure A1.

### Appendix A.1. List of 40 Geons

In Figure A1, we provide a list of 40 geons we have constructed. The label for each geon class represents the four defining shape features, in the order of “axis”, “cross section”, “sweep function”, “termination”, as described in the main paper. We put “na” for the termination when the sweep function is constant. We also distinguish the two termination types “c-inc” and “c-dec” when the sweep function is monotonic. For instance, “c-inc” means that the curved surface is at the end of the increasing sweep function, whereas “c-dec” means that the curved surface is at the end of the decreasing sweep function. As a reference, here is the mapping between the name and the code of 10 geons we used in the 10-geon classification: “Arch”: c_s_c_na; “Barrel”: s_c_ec_t; “Cone”: s_c_m_p; “Cuboid”: s_s_c_na; “Cylinder”: s_c_c_na; “Truncated cone”: s_c_m_t; “Handle”: c_c_c_na; “Expanded Handle”: c_c_m_t; “Horn”: c_c_m_p; “Truncated pyramid”: s_s_m_t.

## Appendix B. Additional Experiments

In this section, we assess the impact of 3D pretraining alone on adversarial robustness. Differing from the experiments described in the main text, we do not perform adversarial training following the 3D pretraining. Instead, we simply fine-tune the model for Geon3D classification. We perform both *L*_2_ and $L_{\infty }$ adversarial attacks for multiple epsilon values using Geon3D-10-BlackBackground. As shown in Figure A2, we see that 3D pretraining itself does not have robustness against adversarial attacks.

## Appendix C. Details of 3D Reconstruction Training

We provide details of the problem setup of 3D reconstruction, following [24].

During training, we render an image, which is then used to minimize the RGB reconstruction loss. To render a pixel of an image observed by a virtual camera, we need to first find the world coordinate of the intersection of the camera ray with the object surface and then map the world coordinate into a RGB color.

Let $u=(u_{1},u_{2})$ be the image coordinate of the pixel we want to render. To find the world coordinates of the intersection, we first parameterize the points along the camera ray $r_{p_{0}\to \left(u_{1},u_{2}\right)}$ by the distance *d* to the camera origin $p_{0}$ as follows:

$$r_{p_{0}\to \left(u_{1},u_{2}\right)}\left(d\right)=R^{T}\left(K^{-1}\left(\begin{array}{c} u_{1}\\ u_{2}\\ d \end{array}\right)-T\right)$$

Here, $R\in R^{3\times 3}$ is a camera rotation matrix, $T\in R^{3}$ is a translation vector, and $K\in R^{3\times 3}$ is a camera intrinsic matrix. In the main paper, we denote $c^{ex}=\left[R,T\right]$ and $c^{in}=K$. Here, *T* is the position of the origin of the world coordinate system with respect to the camera coordinate system. Therefore, the position of the camera origin $p_{0}$ (with respect to the world coordinate system) is $-R^{T}T$.

Then, we solve the following optimization problem:

(A1) $$\mathrm{argmin}\;d\;s.t.\;r_{p_{0}\to \left(u_{1},u_{2}\right)}\left(d\right)\in \Omega$$

where Ω is the set of points *p* in $R^{3}$ such that $f_{\theta }\left(p\right)=0.5$.

To solve for *d*, we start from the camera origin $p_{0}$ and step along the ray until object surface is intersected, which we can determine by evaluating the points along the ray via $f_{\theta }$.

To summarize, we are given a set of object images ${\left\{x_{i}\in R^{H\times W\times 3}\right\}}_{i=1}^{n}$, their corresponding binary object masks ${\left\{m_{i}\in R^{H\times W}\right\}}_{i=1}^{n}$, and extrinsic/intrinsic camera matrices ${\left\{c_{i}=\left(c_{i}^{ex}\in R^{3\times 3}\times R^{3},c_{i}^{in}\in R^{3\times 3}\right)\right\}}_{i=1}^{n}$. Let $U_{0}$ be a set of pixel points which lie inside the ground truth object mask and where the model predicts a depth. $U_{1}$ is a set of points outside the object mask where the model falsely predicts depth. Finally, $U_{2}$ is a set of points inside the object mask where the model does not predict any depth. Then, the objective is as follows:

$$\begin{array}{c} \underset{\phi ,\theta ,\theta^{\prime }}{\arg\;\min}E[\sum_{u\in U_{0}}\left(|\right|{\hat{x}}_{u}-x_{u}{\left|\right|}_{1}+\lambda_{1}L_{\mathrm{normal}}\left({\hat{p}}_{u,c}|z\right))\\ \;+\lambda_{2}\sum_{u\in U_{1}}\mathrm{BCE}\left(f_{\theta }\left({\hat{p}}_{u,c}|z\right),0\right)+\lambda_{3}\sum_{u\in U_{2}}\mathrm{BCE}\left(f_{\theta }\left(p_{\mathrm{rand}(u),c}|z\right),1\right)] \end{array}$$

Here, BCE stands for Binary Cross Entropy loss, and ${\hat{p}}_{u,c}=r_{p_{0}\to u}\left(\hat{d}\right)$, where $\hat{d}$ is the predicted depth, provided as a solution to the optimization problem Equation (A1). The value of $p_{\mathrm{rand}(u),c}=r_{p_{0}\to u}\left(d_{\mathrm{rand}(u)}\right)$, where the value of $d_{\mathrm{rand}}\left(u\right)$ is chosen uniformly randomly on the ray to encourage occupancy for $u\in U_{2}$. ${\hat{x}}_{u}=r_{\theta^{\prime }}\left({\hat{p}}_{u,c}|z\right)$ for $u\in U_{0}$. $z=g_{\phi }\left(x_{i}^{\left(rand\right)}\right)$, where we take a random view $x_{i}^{\left(rand\right)}$ from the same object instance as $x_{i}$.

$L_{\mathrm{normal}}\left(p|z\right)$ is the normal loss, which is a geometric regularizer to encourage smooth object surface. For a point $p\in R^{3}$ and some object encoding *z*, the unit normal vector can be calculated by

$$n_{\theta }\left(p|z\right)=\frac{\nabla_{p}f_{\theta }\left(p|z\right)}{\left|\right|\nabla_{p}f_{\theta }\left(p|z\right){\left|\right|}_{2}}$$

We apply the $l_{2}$ loss to minimize the difference between the normal vectors at *p* and $p^{\prime }$, where $p^{\prime }$ is in a small neighborhood around *p*. Formally,

$$L_{\mathrm{normal}}\left(p|z\right)=\left|\right|n_{\theta }\left(p|z\right)-n_{\theta }\left(p^{\prime }|z\right){\left|\right|}_{2}$$

for a point $p\in R^{3}$.
